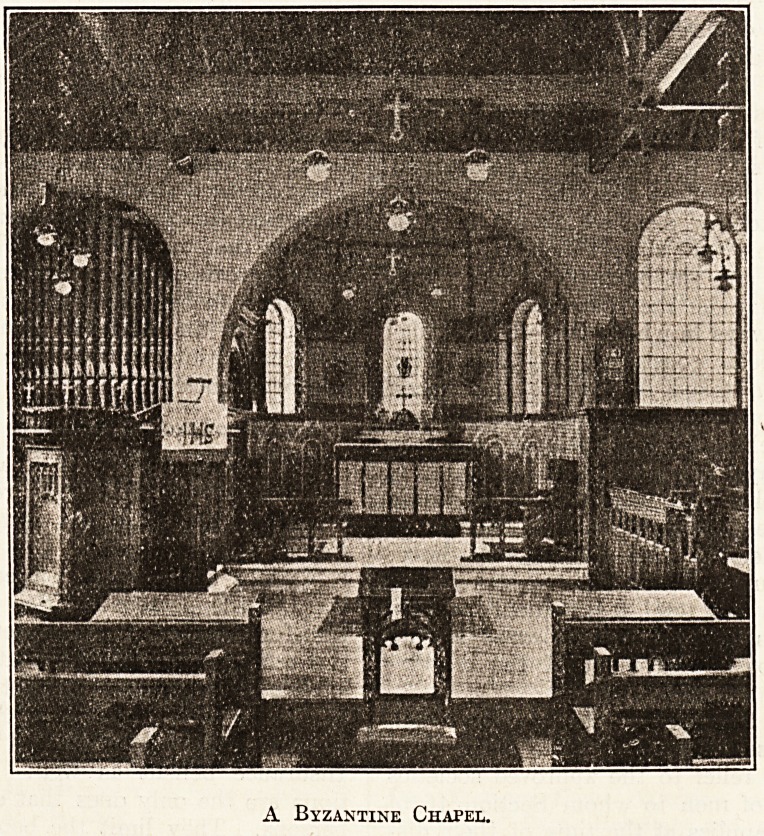# The Chapel of St. Luke the Physician, Camberwell House

**Published:** 1915-03-27

**Authors:** 


### V.—REMARKABLE HOSPITAL CHAPELS.*

(Contributions for this Section Cordially Invited.)

### The Chapel of St. Luke the Physician, Camberwell House.

THIS chapel, which was formally opened at a dedication service held by the Bishop of Woolwich on December 22, 1914, is of unusual magnificence. There were present at the dedication a large gathering of those interested in the treatment of mental disorders, including representatives of the Board of Control and many of the well-known London consultants in psychiatry. In addition to these the Mayor and Corporation of Camberwell attended in state.

A BYZANTINE CHAPEL.

The style is generally Byzantine, this feature being especially shown in the domed apse, which externally is sheathed in copper and surmounted by a Greek cross in gilt. The decoration of the interior of the apse is unusual; from a central point radiate oak beams, and between these the filling is a mastic covered with crushed glass in cerulean blue, picked out with gold stars. This process is Belgian, and has not been employed before in this country. From the centre of the apse hangs an antique sanctuary lamp.

The altar is entirely draped. The beautiful embroidery of the frontals is the work of the Wantage Sisters, and a piece of very fine lace—made by one of the patients—has been incorporated on the super-frontal.

The crucifix, candlesticks, and vases are in bronze, and of Early Italian workmanship. The chalice is also of antique design; it is the gift of the nursing staff. The floor is in mosaic, and the furnishing and panelling are in oak.

There is accommodation for over 400 patients in Camberwell House; but, of course, only a certain proportion of these are able to attend, so that the provision as to seating—150, including the choir—is quite adequate.

The Rev. Dr. O'Brien is the chaplain. Services are held daily, and are attended by the patients and by their friends who come to visit them. The architects were Messrs. Hine and Pegg, of Parliament Street.

* Previous articles appeared on January 23 and 30 and February 13 and 20.

## Figures and Tables

**Figure f1:**